# Psychiatry Outpatients’ Willingness to Share Social Media Posts and Smartphone Data for Research and Clinical Purposes: Survey Study

**DOI:** 10.2196/14329

**Published:** 2019-08-29

**Authors:** Agnes Rieger, Averi Gaines, Ian Barnett, Claudia Frances Baldassano, Mary Beth Connolly Gibbons, Paul Crits-Christoph

**Affiliations:** 1 University of Pennsylvania Philadelphia, PA United States

**Keywords:** social media, smartphone, outpatients, psychiatry, psychotherapy, digital health, mhealth, digital phenotyping, privacy, user preferences

## Abstract

**Background:**

Psychiatry research has begun to leverage data collected from patients’ social media and smartphone use. However, information regarding the feasibility of utilizing such data in an outpatient setting and the acceptability of such data in research and practice is limited.

**Objective:**

This study aimed at understanding the outpatients’ willingness to have information from their social media posts and their smartphones used for clinical or research purposes.

**Methods:**

In this survey study, we surveyed patients (N=238) in an outpatient clinic waiting room. Willingness to share social media and passive smartphone data was summarized for the sample as a whole and broken down by sex, age, and race.

**Results:**

Most patients who had a social media account and who were receiving talk therapy treatment (74.4%, 99/133) indicated that they would be willing to share their social media posts with their therapists. The percentage of patients willing to share passive smartphone data with researchers varied from 40.8% (82/201) to 60.7% (122/201) depending on the parameter, with sleep duration being the parameter with the highest percentage of patients willing to share. A total of 30.4% of patients indicated that media stories of social media privacy breaches made them more hesitant about sharing passive smartphone data with researchers. Sex and race were associated with willingness to share smartphone data, with men and whites being the most willing to share.

**Conclusions:**

Our results indicate that most patients in a psychiatric outpatient setting would share social media and passive smartphone data and that further research elucidating patterns of willingness to share passive data is needed.

## Introduction

### Background

Psychiatry research and clinical assessments often rely on patients’ retrospective reports. Passively collected smartphone and social media data offer a potential alternative to support such measures. Obtaining information on patients' willingness to provide social media and passive smartphone data for research or clinical purposes would inform potential patient recruitment for studies, as well as eventual clinical use. Here, we present a dual focus on researchers and providers’ use of social media and passive smartphone data.

Problems with behavior/symptom self-report have been documented extensively [1]. For example, social desirability is a major concern; participants may be too embarrassed to fully reveal private thoughts/feelings/symptoms or, alternatively, may misrepresent symptoms to ensure study enrollment or treatment continuation. Apart from accuracy concerns, clinical providers have limited time to collect patient information. Calls have been made for a new vantage point and improved assessment methods [2].

A potential alternative, or support, lies in utilizing social media and/or digital device information [3]. Data collected from both patients’ social media and smartphone activity may provide continuous, robust, and ecologically valid insights into mental health. Such insights, which do not rely on retrospective recall, might provide a more nuanced and holistic understanding of behavior and may hold promise for increased accuracy. For example, in clinical care, social media may potentially be used as collateral information to illuminate current stressors, inform diagnoses, and monitor emerging issues. Collateral information, such as input from important people in a client’s life, is commonly used in clinical settings. Moreover, using patients’ own smartphones for data collection, as opposed to providing a phone or bringing patients in for extensive clinical interviews, has the potential for scalability at little additional cost.

Utilizing patients’ social media data and personal smartphones is not without precedent. Many psychiatric care providers are already searching for patients’ electronic communications on the Web, and discussions have begun to explore the implications of utilizing smartphone apps, social media monitoring, etc, in psychiatry [4]. In addition, patients’ phones have already been used for an ecological momentary assessment, in which patients are prompted to answer questions about their current state [5]. In particular, patients’ smartphones have become popular in both research and clinical contexts for monitoring health [6], including an umbrella of mHealth technologies (mobile health) such as activity tracking products that link to the patients’ smartphones [7]. Mental health smartphone apps are on the rise in clinical care and beyond in a rapidly developing marketplace [8].

The potential use of smartphones does not end here; technology exists allowing smartphones to be used for data collection without any user effort, for a field called digital phenotyping. Digital phenotyping is the “moment-by-moment quantification of the individual-level human phenotype in situ using data from smartphones and other personal digital devices” [9]. It includes measures of traditional phone usage (calls and texts), as well as measures that utilize smartphone functions, such as patient movement and activity (derived from a smartphone’s built-in global positioning system [GPS] and accelerometer) [9,10]. The use of digital phenotyping may increase as research groups develop and test smartphone assessments for research measures [9,11] and because of the support expressed by the National Institute of Mental Health for such endeavors in digital health [12]. Social media activity may have similar benefits as it can be mined without patient burden.

Concerns have been expressed that personal digital devices such as smartphones are not widely used by some subgroups of psychiatric patients (eg, those with serious mental illness) [13]. However, recent studies indicate relatively high rates of smartphone usage even among those with schizophrenia [14-16]. A review of 24 studies of health monitoring smartphone apps revealed high levels of retention and acceptability [17]. However, acceptability data on passive smartphone assessment in particular has not been reported. Beyond the logistical concerns, acceptability also involves recognizing concerns about privacy (for a review of research on consumer attitudes toward/reactions to information privacy, see [18]). Particularly relevant to current methods for digital phenotyping data collection, research suggests that even top ranked mental health apps do not accurately convey to users how their personal data may be harvested and shared [19]. Such irresponsibility with sensitive data represents a need to further develop thoughtful research and clinical protocols.

### Objectives

Given the possibility of investigating and incorporating social media and passive smartphone data in psychiatry, several nuanced questions need to be asked about patient acceptability. For example: How comfortable are psychiatric outpatients with the broad spectrum of smartphone data that could be collected for digital phenotyping (ranging from the duration of sleep to how often they answer their phones—an example of data that may be seen as more invasive)? Do publicized breaches in data confidentiality influence the willingness to share personal social media and smartphone information? Would psychiatric outpatients also share such social media and/or passive smartphone data with their provider? It is also useful to consider social media and passive smartphone data collection acceptability simultaneously, as these data sources are complementary.

The purpose of this survey study was to investigate the psychiatric outpatients’ acceptability of social media and passive smartphone data collection (for researchers or clinicians). A survey was administered in a psychiatric outpatient clinic in a large Northeastern city. We asked questions about the participants’ phone use and social media engagement, as well as how comfortable they felt with the researchers and their own therapists having access to data from their smartphones and social media accounts.

The relation of demographic factors (age, race, and sex) to the willingness to share passively collected data (ie, data relevant to digital phenotyping) was also examined. The analysis was exploratory as we did not find extant research on such a relationship. Building upon previous studies that suggested that women are less likely to share private social media information [20], we hypothesized that women would be less likely to share passive data as well. Although relevant research findings are mixed [21], we hypothesized that people of racial minority groups, compared with whites, would be less likely to share, given similar research on health research participation (eg, a study investigating African American participation [22]). Finally, on the basis of research on age and interest in mental health apps [23], we hypothesized that older patients would be less willing to share passive data.

## Methods

### Participants

Participants were patients recruited from a university-based psychiatric outpatient waiting room. Research assistants visited the clinic throughout the day. All people (excluding young children) in the waiting room during a research assistant’s visit were offered the survey. Potential participants who mentioned that they did not own a smartphone were still encouraged to complete the survey. People who completed the survey but did not indicate that they were receiving services from the clinic were excluded as they were not patients (ie, they were waiting with someone who was a patient). Therefore, study participants were all those who (1) returned a survey and (2) indicated that they were patients in the clinic.

The clinic provides psychiatric services to individuals aged 18 years or older. Services include diagnostic evaluations, medication management, and individual and group therapies. The clinic provides specialized treatment for bipolar disorder, treatment-resistant depression, anxiety disorders, substance abuse, psychosis, geriatrics, and medical-psychiatric conditions. Approximately 500 new patients seek services from the clinic each year. Clinic staff includes 15 psychiatric residents, 6 attending physicians, 5 full-time staff psychologists, and 4 part-time psychologists.

The study was approved by the institutional review board; participants provided informed consent. No compensation was provided.

### Measures

The survey was designed in partnership, through discussions among clinical researchers, a digital phenotyping researcher, and the director of outpatient services at the partnering clinic.

#### Demographics/Services

A demographic questionnaire asked for age, sex, race, Hispanic ethnicity, and the services the patient was receiving at the outpatient clinic.

#### Smartphone Ownership

Participants were asked to indicate smartphone ownership, including which model they owned (Apple or Android). Owing to the importance of functioning smartphones for digital phenotyping, we also asked if a participant’s smartphone was in good working condition (participants were asked to select “no” if their phone company frequently shut off their phone or if their phone, for example, did not turn on or was too cracked to read).

#### Smartphone Use

As digital phenotyping relies on consistent device use, we asked if patients usually have their phone with them when they leave home. Responses were: “Yes – I almost never leave my house without my phone,” “In between – I leave my house without my phone about half the time,” and “No – I often leave my house without my phone.” Participants were also asked if their phone served as their alarm clock, if they used their phone before bed, if they looked at their phone upon waking up, and how they communicated via phone (phone calls, texting, Facebook Messenger, Google Chat, KakaoTalk, WhatsApp, WeChat, and other).

#### Social Media Use

Participants were asked to indicate which social media platforms they posted/commented/interacted on. Options were selected by a Web search of currently popular social media options: Ask.fm, Facebook, Instagram, Musical.ly, Pinterest, Reddit, Snapchat, Tumblr, Twitter, and YouTube (if they posted their own videos). “Other” and “I do not use any social media” were also options. We asked the participants to choose from the following options: photos, videos, links (to articles, videos, other peoples’ posts, etc), my mood/feelings, opinions or personal recommendations, reactions (to news, events, other people, etc), important life updates, everyday things that happened in your life, activities, goals/plans for the future, comments/*likes* of other posts, other, and “I never post anything on social media.”

#### Willingness to Share Social Media

Participants were asked if they would share social media posts with their therapist if their therapist was concerned about how they were doing. If they would, they were asked what they would share: “Only the postings that I make public,” “Both my public and my private postings,” and “I would pick-and-choose posts from both my public and private postings.”

#### Publicized Privacy Breach Influence

We selected the Cambridge Analytica scandal as an example of a publicized social media data privacy breach as the media story was a particularly publicized example at the time of survey administration. A brief description was provided: “Recently, Facebook has been in the news for its use of personal data from Facebook Accounts through a company, Cambridge Analytica.” Participants were asked if this privacy violation made them more hesitant about “your smartphone data being collected by the university?” “sharing your smartphone data with your therapist (as part of a research study)?” and “sharing your social media with your therapist (as part of a research study)?”

#### Willingness to Share Passive Smartphone Data

Patients who had a smartphone were asked to specify what parameters would be acceptable to them to be collected via a smartphone app in the context of a research study. One category was information collected using GPS: “amount of time you spend at home,” “amount of time during your day you spend not moving,” “distance you travel,” and “maximum distance you travel from your home.” Patients were asked if it was acceptable to collect information on how long they sleep each day, as assessed by tracking how long the phone screen is turned on/off. Patients were also asked if it would be acceptable for researchers to collect the number of texts and calls sent/received, the length of texts and calls sent/received, and how often the phone is answered. Finally, participants were asked if it would be acceptable for their therapist to also have access to this information. The sum of the digital phenotyping–relevant data items that a participant was willing to share was calculated.

### Statistical Analyses

Questions were summarized descriptively with percentages or means, as appropriate. Information relevant to passive smartphone data collection was summarized for the sample of patients who specifically indicated that their phone was in good working condition. Information relevant for social media activity was summarized for those who had at least one social media account, and information relevant to sharing data with therapists was summarized for those who were receiving talk therapy. A multiple regression was conducted to evaluate the relation of age, sex, and race to the willingness to participate in a digital phenotyping study. Race was dichotomously coded as white and everyone who selected another race.

## Results

### Characteristics of the Sample

A total of 238 patients agreed to participate and returned a survey. Characteristics of the sample are presented in Table 1. Ages of the participants ranged from 18 to 84 years; the average age was 39.4 (SD 15.7) years.

### Smartphone Ownership and Use

A high percentage of patients (219/235, 93.2%) owned a smartphone. Most (201/214, 93.9%) indicated that they had a working smartphone (24/238, 10.1% did not respond to the working smartphone item; 84.4%, 201/238, of the full sample thus had a working smartphone). Considering those with a working smartphone, 69.8% (139/199) owned an Apple model, whereas 30.2% (60/199) owned an Android model. Smartphone use characteristics are reported in Table 2; modes of mobile phone communication are presented in Table 3.

### Social Media Use

Table 4 gives the percentages of social media activities for those who indicated that they used at least one social media platform (N=199).

**Table 1:** Patient demographic characteristics.

Characteristics^a^		Participants, n (%)^b^
Sex, female (N=198)		141 (71.2)
Ethnicity, Hispanic (N=231)		17 (7.4)
**Race (N=238)**		
	American Indian or Alaska Native	5 (2.1)
	Asian	6 (2.5)
	Black or African American	56 (23.5)
	Native Hawaiian or Pacific Islander	1 (0.4)
	Others or unknown	10 (4.2)
	White	167 (70.2)
**Services (N=238)**		
	Only medication management	78 (32.8)
	Only talk therapy	37 (15.5)
	Receiving both services	123 (51.7)

^a^Percentages for race/ethnicities are greater than 100% as participants were welcome to select more than one response.

^b^Results were summarized as the total count of people who indicated the answer listed in the table over the total count of people who answered the specific question (ie, participants who skipped a question were excluded from the analysis of that particular question; hence, the denominators fluctuate).

**Table 2:** Phone use relevant to leaving home and sleep habits.

Characteristics	Participants, n (%)^a^
Never leave home without phone (N=201)	196 (97.5)
Sometimes leave home without phone (N=201)	4 (2.0)
Often leave home without phone (N=201)	1 (0.5)
Use phone as alarm clock (N=199)	157 (78.9)
Look at phone before bed (N=198)	173 (87.4)
Look at phone when they wake up (N=183)	162 (88.5)

^a^Results were summarized as the total count of people who indicated the answer listed in the table over the total count of people who answered the specific question (ie, participants who skipped a question were excluded from the analysis of that particular question; hence, the denominators fluctuate).

**Table 3:** Modes of mobile phone communication (N=201).

Means of communication on mobile device	Participants, n (%)^a^
Phone calls	193 (96.0)
Texting	196 (97.5)
Facebook Messenger	109 (54.2)
Google Chat	16 (8.0)
KakaoTalk	1 (0.5)
WhatsApp	44 (21.9)
WeChat	1 (0.5)
Others	52 (25.9)

^a^Results were summarized as the total count of people who indicated the answer listed in the table over the total count of people who answered the specific question (ie, participants who skipped a question were excluded from the analysis of that particular question; hence, the denominators fluctuate).

**Table 4:** Patient social media use (N=199).

Social media platforms used		Outpatients who use social media, n (%)^a^
**Social media platforms used**		
	Ask.fm	1 (0.5)
	Facebook	164 (82.4)
	Instagram	123 (61.8)
	Musical.ly	4 (2.0)
	Pinterest	40 (20.1)
	Reddit	28 (14.1)
	Snapchat	56 (28.1)
	Tumblr	19 (9.5)
	Twitter	58 (29.1)
	YouTube	25 (12.6)
	Other	13 (6.5)
**Content of social media posts**		
	Photos	152 (76.4)
	Videos	77 (38.7)
	Links (to articles, videos, other peoples’ posts, etc)	105 (52.8)
	Mood/feelings	51 (25.6)
	Opinions or personal recommendations	63 (31.7)
	Reactions (to news, events, other people, etc)	80 (40.2)
	Important life updates	72 (36.2)
	Everyday things that happened in life	39 (19.6)
	Activities	53 (26.6)
	Goals/plans for the future	25 (12.6)
	Comments/*likes* of other posts	123 (61.8)
	Others	13 (6.5)
	Never post on social media	13 (6.5)

^a^Results were summarized as the total count of people who indicated the answer listed in the table over the total count of people who indicated having at least one social media account.

Of those who had a social media account and were receiving talk therapy, 74.4% (99/133) indicated that they would be willing to share their social media posts with their therapists, if their therapist were concerned about how they were doing. In a follow-up question, 20.2% (19/94) indicated that they would only share the postings that they make public, 53.2% (50/94) would share both public and private posts, and 26.6% (25/94) would pick-and-choose public and private posts.

### Willingness to Share Passive Smartphone Data

Table 5 gives the full reporting of the patients’ willingness to share passively collected data parameters.

**Table 5:** Passive smartphone data participants are willing to share with researchers.

Data comfortable sharing with researchers	Participants, n (%)^a^
Amount of time spent at home (N=201)	100 (49.8)
Amount of time during the day spent not moving (N=201)	102 (50.7)
Distance traveled (N=201)	113 (56.2)
Maximum distance traveled from home (N=201)	93 (46.3)
How long one slept each day (N=201)	122 (60.7)
Number of texts sent (N=201)	105 (52.2)
Length of texts sent (N=201)	85 (42.3)
Number of texts received (N=201)	98 (48.8)
Length of texts received (N=201)	82 (40.8)
Number of calls made (N=201)	101 (50.2)
Length of calls made (N=201)	89 (44.3)
Number of calls received (N=201)	99 (49.3)
How often one answers their phone (N=201)	94 (46.8)
Length of calls received (N=201)	88 (43.8)
Willing to share same data with therapist (N=184)	123 (66.8)

^a^Results were summarized as the total count of people who indicated the answer listed in the table over the total count of people who indicated that they had a working smartphone.

### Demographic Correlates of Willingness to Participate in a Passive Smartphone Assessment Study

Multiple regression analyses on those with a working smartphone revealed that sex and race, but not age, were significantly associated with the composite variable measuring how many types of passive smartphone data patients would be willing to share in a research study. Partial correlations were as follows: age, *r*_p_=0.03 (*P*=.69); sex, *r*_p_=−0.16 (*P*=.04); and race, *r*_p_=−0.16 (*P*=.05). Men (mean 8.6, SD 5.9) were more willing to share more information than were women (mean 6.3, SD 5.6). People who were of any race other than white (mean 5.2, SD 4.8) were less willing to share more information than whites (mean 7.4, SD 6.0). The results of analyses predicting the willingness to share individual parameters from the demographic variables yielded similar results as found with the summary score.

### Publicized Privacy Breach Influence

Of the patients with a working smartphone, 30.4% (59/194) indicated that Facebook’s Cambridge Analytica privacy breach made them more hesitant about researchers collecting their passive smartphone data. Similarly, 27.3% (35/128) of those who had a working smartphone and who were in talk therapy believed that the privacy breach made them more hesitant about their therapist receiving that data. Considering the outpatients who indicated that they used at least one social media platform and who were receiving talk therapy at the clinic, 23.4% (30/128) were more hesitant about their therapist receiving that data.

## Discussion

### Principal Findings

Psychiatry researchers and practitioners have an invested interest in outpatients’ willingness to share certain parts of their lives and in the various ways in which that *data*, broadly speaking, may be collected and shared. From allowing a researcher to collect information on how long they sleep as measured by phone activity to permitting a concerned therapist to view their social media, the sharing of passive smartphone and social media data by patients presents a potentially well-supplied opportunity. To date, research studies have not explicitly investigated outpatients’ acceptability of specific passive smartphone parameters. One study that did inquire about specific parameters was qualitative and more broadly interested in the affective and thought response to digital phenotyping [24].

Our survey results indicate that research involving the collection of social media and passive smartphone data with patients in psychiatric treatment is acceptable, but not all patients are willing to share such data with their therapist or with researchers. As a starting point, we assessed the willingness to share social media and smartphone data without a particular research purpose or proposed design. More targeted surveys can probe the willingness to share within specific contexts—some of which might generate greater or lesser willingness than we found. Within our sample of outpatients in therapy and with social media, 74.4% (99/133) were willing to share social media data with their therapists if their therapists were concerned, and 40.8% (82/201) to 60.7% (122/201) were willing to share various passive smartphone parameters with the researchers. We did not ask if the patients would routinely share their social media information with a therapist (regardless of any ongoing concern by the therapist about how the patient was doing). Presumably, even fewer people would share all their social media content all the time.

At 93.2%, the overwhelming majority of outpatients indicated that they owned a smartphone. Although this finding is a higher rate than previously estimated for the US population as a whole [23,25], considering that 95% of our participants were aged between 18 and 64 years, our finding is more comparable with the national data (ie, Pew Research has reported a national rate of about 85% for this age group [25]). It is important to note that our reporting of smartphone-relevant data sharing is limited to those who have a smartphone. If it is the case that people without a smartphone were, for any reason, less likely to participate, then this situation would not affect the results, as those without a smartphone were excluded from the analyses. The methods of recruitment can always bias results. In our case, patients who mentioned that they did not have a smartphone were always requested to continue with the survey and indicate on the survey that they did not own a smartphone.

Considering smartphone ownership, we went one step further to specifically ask if the participants’ smartphones were in good working condition (ie, that they were usable and equipped with a consistent phone plan). Not only did the outpatients frequently own smartphones in good working condition, they frequently took those phones with them when they left their homes (an especially important consideration when planning on using smartphones). Collecting passive data from smartphones is therefore theoretically possible in this population. However, approximately 40% to 60% of the patients (depending on the parameter) were willing to share specific passive smartphone data with researchers. Overall, 66.8% (123/184) were willing to allow a therapist to have access to the same information that they would share with a researcher. Researchers and providers interested in clinical applications of passive smartphone data will need to consider that about one-third of the patients would hesitate to share such information with their therapists.

Contributing to this level of nonsharing, over one-fourth of our outpatient sample reflected that they were more hesitant about sharing passive smartphone data as a result of Facebook’s Cambridge Analytica scandal. We chose to inquire specifically about the Cambridge Analytica media story as, when the survey was administered, it was a well-publicized example of a privacy/security misuse. Although 30.4% (59/194) of the people shared that it was an issue, it is unclear if the issue had not been brought up, whether the patients themselves would have spontaneously identified this example of a media privacy concern. Identifying the specific media story ourselves may have resulted in the scandal being perceived as a greater barrier than what would have actually occurred in a study that actually used social media and passive smartphone data.

It may also be the case that some survey participants are particularly attuned to privacy concerns related to health/ apps, either by a propensity to worry about privacy or through prior knowledge of private data breaches. Even beyond the Cambridge Analytica breach, health-related apps found in public app stores may have extensive data sharing concerns, shrouded in a lack of transparency [19]. Future survey research should include items that explore how much a participant is already mindful of their health data sharing. Indeed, we would have also done well to inquire about how concerned people were with the content of what would be shared through an app (ie, some people may be aware that they display behavior on their phones that is more, perhaps, noteworthy than others).

Although still keeping in mind that, by asking about Cambridge Analytica, we chose a specific example connected to 1 specific social media platform (Facebook), we posit that our finding suggests that publicized data scandals in general may be associated with outpatients’ willingness to participate in relevant research. The broader issue at hand, then, is that data handling in the corporate sphere, as well as in the academic research sphere, has ramifications that influence the other. Dealing with personal social media data (and, considering our interests, smartphone data as well) is a privilege that should not be taken lightly.

We examined the overall willingness to share passive smartphone data based on the demographic variables of sex, age, and race. We did not locate previous studies that conducted these analyses. Our exploratory findings therefore need to be confirmed in future studies and, if confirmed, the potential reasons for any differences should be examined.

Our finding that women may be more hesitant to share passive data is consistent with previous studies suggesting that women are less likely to share private social media information [20]. In our exploratory analyses, owing to our sample sizes, we compared whites with everyone who did not fall under that category (a diverse group of people who selected anything other than white). Our exploratory finding might build on extant research exploring potential differences by race in willingness to participate in research, broadly. Such existing research most often involves comparisons of African Americans and whites and indicates that African Americans may be more hesitant to participate in health research [22], though further studies questioning the assumption that willingness might differ by race is emerging [21]. We encourage more specific research in this area.

### Limitations

Several limitations of this survey require notice. People who anonymously indicate that they would share data may not actually do so when presented with an imminent opportunity. Demographic predictors of the willingness to share passive smartphone data were conducted on an exploratory basis and therefore would need replication. Sample sizes were not large enough to examine the influence of specific identities on the willingness to share. We were interested quite broadly in outpatients; we did not collect data on specific mental health concerns. It is unknown if recruiting participants in the waiting room influenced results.

### Conclusions

Our results indicate that work seeking to collect social media and passive data in a psychiatric outpatient sample is largely acceptable for outpatients. About half of our sample was willing to share data that, for some, may seem particularly invasive and unacceptable to patients for researchers and providers to collect. However, our results indicate that the work involving social media use in this population may be challenging owing to a lack of engagement with multiple platforms and that research involving passive data collection from smartphones may call for targeted recruitment strategies.

## Appendix

Multimedia Appendix 1 To be used in publication: the original survey administered to participants.

## Abbreviations

GPS: global positioning system
