# Supplementary material for: Psychiatry Outpatients’ Willingness to Share Social Media Posts and Smartphone Data for Research and Clinical Purposes: Survey Study
Source: JMIR Form Res. 2019 Aug 29;3(3):e14329. doi: 10.2196/14329 (PMC6754680; doi:10.2196/14329)
Supplement: Multimedia Appendix 1 [file formative_v3i3e14329_app1.pdf]

Patient Questionnaire

<u>If you have already completed this form, please do not complete it again.</u>

1) **Race/ethnicity:** (please check all that apply)
   - ☐ American Indian or Alaska Native
   - ☐ Asian
   - ☐ Black or African American
   - ☐ Native Hawaiian or Pacific Islander
   - ☐ White
   - ☐ Other or unknown

2) **Are you Hispanic or Latinx?**
   ☐ Yes ☐ No

3) **Age:** ___ years old

4) **Sex:** ☐ M ☐ F

5) **At this time, at Penn Behavioral Health, what services are you receiving?**
   ☐ Medication management ☐ Talk therapy ☐ Both

6) **Are you interested in receiving talk therapy at this clinic?**
   ☐ Yes ☐ No

7) **Do you own a smartphone?** "Smartphones" are phones that can use your location (GPS), download apps, and connect to the Internet.
   ☐ Yes ☐ No

   If yes: **Is your smartphone apple or android?**
   - ☐ Apple (iPhone)
   - ☐ Android (Samsung Galaxy, HTC, Motorola, etc.)

   If yes: **Is your phone in good working condition?** Please check "No" if your phone is frequently shut off by the phone company, or if your phone does not work well (e.g. if your phone does not turn on or if your screen is too difficult to read due to cracks).
   ☐ Yes ☐ No

8) **Do you usually have your phone with you when you leave home?**
   - ☐ Yes – I almost never leave my house without my phone.
   - ☐ In between – I leave my house without my phone about half the time.
   - ☐ No – I often leave my house without my phone.

9) **Do you use your phone as an alarm clock to wake up?**
   ☐ Yes ☐ No

10) **Do you look at your phone before bed?**
   ☐ Yes ☐ No

11) **When you wake up?**
   ☐ Yes ☐ No

12) **How do you use your phone for communicating with family, friends, or for work?** Please check all that apply.
   - ☐ Phone calls
   - ☐ Texting
   - ☐ Facebook messenger
   - ☐ Google chat
   - ☐ Kakao talk
   - ☐ WhatsApp
   - ☐ WeChat
   - ☐ Other

Patient Questionnaire

13) **Which social media do you use?** Please check all of the social media that you post/comment/interact on, **and circle your one favorite** social media.

- ☐ ask.fm
- ☐ Facebook
- ☐ Instagram
- ☐ musical.ly
- ☐ Pinterest
- ☐ reddit
- ☐ SnapChat
- ☐ Tumblr
- ☐ Twitter
- ☐ YouTube (only select if you post your own videos)
- ☐ Other

- ☐ I do not use any social media

14) **What do you post on social media?** Please check all that apply.

- ☐ Photos
- ☐ Videos
- ☐ Links (to articles, videos, other peoples' posts, etc.)
- ☐ My mood/feelings
- ☐ Opinions or personal recommendations
- ☐ Reactions (to news, events, other people, etc.)
- ☐ Important life updates
- ☐ Everyday things that happened in your life
- ☐ Activities
- ☐ Goals/plans for the future
- ☐ Comments/ "likes" of other posts
- ☐ Other

- ☐ I never post anything on social media

15) **Would you be willing to share your social media posts (e.g. Facebook, Twitter, Instagram, etc.) with your therapist if your therapist was concerned about how you were doing?**

☐ Yes ☐ No

If yes: **What social media content would you be willing to share with your therapist?**

- ☐ Only the postings that I make public.
- ☐ Both my public and my private postings.
- ☐ I would pick-and-choose posts from both my public and private postings.

Patient Questionnaire

16) Recently, Facebook has been in the news for its use of personal data from Facebook Accounts through a company, Cambridge Analytica. **Does this privacy violation make you more hesitant about:**

your smartphone data being collected by the University of Pennsylvania?

◻ Yes ◻ No

sharing your smartphone data with your therapist (as part of a research study)?

◻ Yes ◻ No

sharing your social media with your therapist (as part of a research study)?

◻ Yes ◻ No

If you don't have a smartphone, please skip #17 and #18, and turn over the page to continue the survey.

---

17) Below is a list of information that a research app has the ability to collect from your smartphone. **Please select the information that you would be okay with the app collecting.** This information would be collected confidentially and shared only with professional researchers at the University of Pennsylvania.

<u>Information collected using your phone's GPS:</u>

- ☐ Amount of time you spend at home
- ☐ Amount of time during your day you spend not moving
- ☐ Distance you travel
- ☐ Maximum distance you travel from your home

<u>Information collected by tracking how long your phone screen is on/off:</u>

- ☐ How long you sleep each day

<u>Information collected about your communication:</u>

- ☐ Number of texts you send
- ☐ Length of the texts you send
- ☐ Number of texts you receive
- ☐ Length of the texts you receive
- ☐ Number of calls you make
- ☐ Length of the calls you make
- ☐ Number of calls you receive
- ☐ How often you answer your phone
- ☐ Length of the calls you receive

18) Would you be okay with your therapist also having access to that information?

☐ Yes ☐ No
